# Shifting the social determinants of food insecurity during the COVID-19 pandemic: the Australian experience

**DOI:** 10.1007/s12571-022-01318-4

**Published:** 2022-09-17

**Authors:** Christina Zorbas, Jennifer Browne, Alexandra Chung, Anna Peeters, Sue Booth, Christina Pollard, Steven Allender, Anna Isaacs, Corinna Hawkes, Kathryn Backholer

**Affiliations:** 1 Global Centre for Preventive Health and Nutrition, School of Health and Social Development, Faculty of Health, Institute for Health Transformation, Deakin University, Geelong, Australia; 2 School of Public Health and Preventive Medicine, Monash University, Melbourne, Australia; 3 College of Medicine and Public Health, Flinders University, Adelaide, Australia; 4 School of Population Health, Faculty of Health Sciences, Curtin University, Perth, Australia; 5 Centre for Food Policy, School of Health Sciences, Division of Health Services Research and Management, City, University of London, London, United Kingdom

**Keywords:** COVID-19, Health equity, Social determinants, Food insecurity, Food policy, Social policy

## Abstract

We aimed to explore experiences of government-led actions on the social determinants of food insecurity during Australia’s COVID-19 pandemic response (which included novel, yet temporary, social protection measures to support Australians facing hardship during state-wide lockdowns). During November–December 2020, we conducted in-depth interviews with 24 Victorians who received government income support (prior to COVID-19) and the temporary COVID-19 specific payments. Interviews were guided by a theoretical understanding of the social determinants of health and health inequities, which we aligned to the social policy context. Data were audio-recorded, transcribed, inductively coded, categorised and thematically analysed. Our sample included mostly women (n = 19) and single parents (n = 13). Interviews reflected four key themes. Firstly, participants described ‘battles all around them' (i.e., competing financial, health and social stressors) that were not alleviated by temporary social policy changes and made healthy eating difficult to prioritise during the pandemic. Secondly, housing, income, job, and education priorities rendered food a lower and more flexible financial priority – even with 18 participants receiving temporary income increases from COVID-19 Supplements. Thirdly, given that food remained a lower and more flexible financial priority, families continued to purchase the cheapest and most affordable options (typically less healthful, more markedly price discounted). Finally, participants perceived the dominant public and policy rhetoric around income support policies and healthy eating to be inaccurate and shaming – often misrepresenting their lived experiences, both prior to and during COVID-19. Participants reported entrenched struggles with being able to afford basic living costs in a dignified manner during COVID-19, despite temporary social protection policy changes. To reduce inequities in population diets, a pre-requisite to health, all stakeholders must recognise an ongoing responsibility for adopting long-term food and social policies that genuinely improve lived experiences of food insecurity and poverty.

**Supplementary Information:**

The online version contains supplementary material available at 10.1007/s12571-022-01318-4.

## Background

In 2020, 2.37 billion people worldwide (320 million more than in 2019) faced food insecurity (FAO et al., 2020). Inadequate access to safe and nutritious food is a key risk factor for weight gain and diet-related diseases in many high-income countries (Banerjee et al., 2020; Moradi et al., 2019). The burden of diet-related disease is greatest among people experiencing social and/or economic disadvantage due to low income, low education, occupation status, and/or ethnicity (Australian Institute of Health and Welfare, 2019; Backholer et al., 2016). The inequitable distribution of dietary risks and diet-related diseases reflects the inequitable conditions in which people are born, work, live, and age (i.e., the social determinants of health and health inequities) (Friel et al., 2015; World Health Organization, 2008). These daily living conditions are shaped by the ways in which our societies are governed through social, economic, public, and health policies that determine opportunities to purchase and consume healthy diets. For example, in some countries, social protection policies can directly supplement incomes, and therefore the food budgets, of those who cannot fully engage in work because of illness, age, caring responsibilities, inadequate job opportunities and unemployment (Phillips et al., 2021).

The global COVID-19 pandemic has had major and almost instantaneous impacts on the social determinants of population diets, diet-related health, and health inequities (Paremoer er al., 2021). To suppress COVID-19 within the Australian community, national and state governments enforced extensive regulatory measures, including several lockdowns. From March 2020 to November 2021, the Australian state of Victoria implemented lengthy lockdown periods (in excess of 200 days) and periods of quarantine and isolation for multiple communities; resulting in business closures (temporary and permanent), a rise in unemployment, remote learning from home, restrictions on most forms of social participation, and physical distancing between people (Australian Bureau of Statistics, 2021; Mclean & Huf, 2020). To support the growing number of Australians negatively affected by the COVID-19 pandemic, the Australian Government temporarily implemented new, and expanded existing, social protection policies (Australian Council of Social Services, 2018).

Australia’s COVID-19 policy changes included, but were not limited to, supplemented income support payments (i.e., doubling of existing income support for persons experiencing unemployment through the ‘JobSeeker’ scheme; benefits provided to employers to retain employees through the ‘JobKeeper’ scheme), free childcare, temporary rent relief grants, and prohibitions on landlords increasing rent (Table S1). At its peak, the income support provided through JobSeeker could be considered radical (albeit temporary), constituting fortnightly supplement payments of $AUD 550 in addition to the original income support rate for unemployed persons (i.e., ‘Newstart’: $AUD 546 per fortnight) (Parliament of Australia, 2020). Indeed, the original income support rate in Australia had not been increased since 1994 (Bradbury & Hill, 2021) (while during the same period, the consumer price index increased by > 90% (Australian Government: Australian Tax Office, 2022)), was below absolute and relative poverty lines (Melbourne Institute: Applied Economic & Social Research, 2020), and was the lowest rate in the Organisation for Economic Co-operation and Development (OECD, 2022). The COVID-19 policy changes were in effect at various timepoints between March 2020 and March 2021, with the JobSeeker Supplement gradually reduced from September 2020 (and various social supports introduced during lockdown periods following March 2021) Australian Government: Economic Response to the Coronavirus, 2020; (Parliament of Australia, 2020).

Currently, the highest poverty rates in Australia occur among households receiving government income support, particularly single-parent households (Phillips & Narayanan, 2021). As of April 2021, it was estimated that 4.2 million Australians were living in poverty, including 750,000 children; estimates that exceed those prior to COVID-19 (3.7 million Australians – including 624,000 children) (Phillips & Narayanan, 2021). A survey of 955 people receiving the COVID-19 Supplement payment early in the pandemic (May 2020) reported that increases to income support payments resulted in a 56% decrease in meal skipping (compared to the original payments), with 93% of respondents also reporting being able to afford eating more fresh fruits and vegetables (Australian Council of Social Services, 2020). Evidence also found that the COVID-19 specific increase in income rendered healthy diets affordable for families receiving low incomes for the first time (costing about $AUD 600 or 20% of the supplemented household income, per fortnight (Lewis & Lee, 2020)). These results suggest that increases to income support payments may be effective in reducing widening inequities in diet-related health (Australian Council of Social Services, 2020; Gearon et al., 2020; World Health Organization, 2008).

Recommendations by leading health organisations indicate that sustained, evidence-based government policies are critical to reduce inequities in healthy eating – with actions to reduce poverty being core (London's Child Obesity Taskorce. Greater London Authority, 2019; Saunders et al., 2017). Nonetheless, our previous research has shown that national governments do not have the tools or adequate commitment to achieve these public health imperatives (Chung et al., 2021; Zorbas et al., 2020a, 2021). Equity-oriented policy progress is likely to continue to be hindered by the inadequate representations of the voices and values of those experiencing social and economic exclusion in policy processes, research and advocacy efforts (Browne et al., 2019; Centre for Public Impact - A BCG Foundation, 2020). Research focused on understanding and elevating the voices of people with first-hand experiences of disadvantage is termed lived experience research – a field which critically endeavours to challenge accepted ways of knowing, inequities, and systemic power imbalances (Nemours Children’s Health System, Nemours National Office of Policy & Prevention, 2020). To date, research has explored lived experiences of food-focused approaches to addressing food insecurity, including experiences of the Supplemental Nutrition Assistance Program in the US (Chiappone et al., 2019; Gosliner et al., 2020), food charities (Booth et al., 2018; Middleton et al., 2018), and individual-level coping strategies (Graham et al., 2018; Middleton et al., 2018). In comparison, few studies (and none in Australia) have focused on the lived experiences of food security after receiving improved social policy supports. Despite general acceptance that social policies are required to address food insecurity (Pollard & Booth, 2019), actions outside of health or food systems have seldom occurred in the real-world, especially in Australia – rendering them difficult to study (Friel et al., 2015). Thus, the rapid and temporary changes to social policies to reduce vulnerability during the COVID-19 pandemic in Australia provided a unique opportunity to address this gap.

The aim of this study was to understand how rapid and temporary changes to the social determinants of health (via government-led actions, implemented in direct response to the COVID-19 pandemic) affected experiences of food security and wellbeing among Australians in the state of Victoria who received social supports prior to COVID-19. Participants were asked about their previous experiences with government supports and how their experiences may have changed with the new and seemingly improved COVID-specific social policies (described above). We hypothesised that the additional social supports would enable participants to better prioritise healthy diets. Whilst published research has explored experiences of food policies and food insecurity, this study adds to our understanding of the short-term impacts of social policy changes.

## Methods

This study was reported according to the COnsolidated criteria for REporting Qualitative research (COREQ) (Tong et al., 2007).

### Study design

Our study begins with a social constructionist epistemology – whereby an individual’s views are used to construct meaning based on the intersection between personal experiences and/or perspectives and social interactions (Berger & Luckmann, 1966). Our critical qualitative descriptive design focused on capturing and amplifying collective experiences to inform and advocate for policy changes (Kincheloe et al., 2017). Due to the COVID-19 restrictions across the state of Victoria (described above), our methods were limited to in-depth telephone or video teleconference interviews.

### Sampling and recruitment

We purposively sampled 24 Victorian adults (also referred to as Victorians herein), representing the main grocery shoppers from low-income households (defined using national indicators for financial distress (Australian Bureau of Statistics, 2017)) who were receiving government income support prior to COVID-19 and COVID-19 specific government supports. Experience and evidence indicates that 20 interviews are usually adequate to produce rich data from a similar group of participants, and that a few extra interviews should be conducted to confirm this (Vasileiou et al., 2018). Rather than recruiting for theoretical saturation, participants were recruited to enable preliminary investigations into how lived experiences might vary across different households. During recruitment, families with children were prioritised; however, we also recruited a sub-sample of households without children to explore the transferability of our findings (n = 6 households). We additionally aimed to achieve equal participation across Metropolitan and Regional Victoria – noting the relatively higher levels of socioeconomic disadvantage in regional compared to metropolitan areas (Australian Institute of Health & Welfare, 2019).

We initially planned to recruit participants through local councils and community organisations, but this was not possible due to the lockdown restrictions in Victoria. We therefore employed a local recruitment company with access to an online panel of Victorians that have volunteered to be contacted to participate in research. Potential participants were invited to take part in the study before being asked a series of demographic screening questions (receipt of government income support schemes, household composition, work status, household income, indicators of financial distress; Table S2) to enable purposive sampling.

### Data collection

Semi-structured interviews were conducted via telephone or video teleconference (Zoom). Participant sociodemographic data (sex, age, household/family composition, Aboriginal or Torres Strait Islander Status, education level, occupation, household income, receipt of social protection payments (Y/N and type)) were collected at the beginning of interviews. Our semi-structured interview guide included questions about food experiences during the COVID-19 pandemic, the influence of the COVID-19 specific social policy changes on these experiences (which were mapped against our existing theoretical understanding of the social determinants of diets and health inequities), and recommended policy actions and actors (Table S3). A participant-centred approach was taken during the interviews, recognising the importance of building rapport and allowing the authentic voices of participants to guide our understanding of where food stands as a priority in their broader life experiences (Prior et al., 2020).

The interview guide was reviewed by multiple members of the research team, all with extensive qualitative and/or health equity research experience. One trained qualitative researcher (CZ) conducted, and audio recorded each interview (mean duration of 52 min except for two shorter interviews where participants were reluctant to provide in-depth answers, < 20 min).

### Data analysis

Our analytic approach was guided by Braun and Clarke’s (2006) six phases of thematic analysis (adapted to facilitate a team-based analysis) (Braun & Clarke, 2006). Following familiarisation with the interview data and transcripts, interviews were initially coded inductively in a block-by-block manner by the lead researcher (CZ) using NVivo 12. Codes were constantly compared (including exporting, tabulating, and reorganising codes) and iteratively categorised into sub-themes and themes. A second researcher (JB) independently cross-coded a sample of transcripts (n = 3, ⁓10%) and worked with the lead researcher to develop a thematic framework (Table 1) – which was subsequently used to code the remaining interview transcripts, whilst allowing flexibility for new codes and themes to be generated. The final themes represent summaries of participants’ experiences with food and rapid (yet temporary) changes to the social determinants of diets during the COVID-19 pandemic in Victoria, Australia. All themes were reviewed and confirmed by the research team. Quotes are presented to illustrate and exemplify the final themes and sub-themes.

**Table 1:** Interviewee characteristics (n = 24 Victorians receiving government income support during COVID-19)

**Gender (Female)**	*n*	19
**Age**	*Mean (SD)*	41 (10)
**Metro; Regional**	*n*	11; 13
**Area-level disadvantage, IRSD**^**a**^	Q1, *n*	5
	Q2, *n*	7
	Q3, *n*	5
	Q4, *n*	5
	Q5, *n*	2
**Aboriginal or Torres Strait Islander**	*n*	2
**Born in Australia**	*n*	22
**Speak a language other than English at home**	*n*	1
**Highest level of education**	High school, *n*	8
	Certificate III, *n*	2
	Diploma/Advanced diploma, *n*	8
	Bachelor degree, *n*	3
	Graduate diploma/Postgraduate degree, *n*	3
**Employment status**	Unemployed, *n*	13
	Working part time, *n*	6
	Casual work, looking for more hours, *n*	1
	Carer/home duties, *n*	4
**Household composition**	2 parent family, oldest child aged under 13, *n*	1
	2 parent family, oldest child aged 13 and over, *n*	4
	Single, shared custody of child aged 13, *n*	1
	Single parent household with kids, *n*	10
	Single with children living with parents, *n*	2
	Single with no children, *n*	3
	Couple with no children, *n*	1
	Empty nester, *n*	2
**Self-reported household income**	< $AUD 25,000, *n*	10
	< $AUD 25,000–50,000, *n*	14
	Single household income, *n*	19
	Double household income, *n*	5
**Number of ABS financial distress indicators**^**b**^** (SD)**	*Mean (SD)*	6 (2.1)
**Housing**	Private rental, *n*	10
	Owned with mortgage, *n*	3
	Owned (with legal issues), *n*	1
**Government income support received prior to COVID-19**	Parenting payment, *n*	7
	Carers payment, *n*	2
	NewStart (former unemployment support scheme), *n*	13
	Did not receive/unclear, *n*	2
**Government income support received since COVID-19**	JobSeeker (current unemployment support scheme), *n*	17
	JobKeeper (COVID-19 job support scheme), *n*	5
	Other COVID-19 supplement, *n*	2
**Overall increase or decrease in income during COVID-19**	Increase, *n*	18
	Decrease, *n*	6
**COVID-19 rent relief grant received**	*n*	1
**Free childcare received**	*n*	4
**Child did remote learning from home during COVID-19**	*n*	16

^a^Index of Relative Socio-economic Disadvantage (IRSD; Q1: most disadvantaged, Q5: least disadvantaged) (Socio-Economic Indexes for Areas. Australian Bureau of Statistics. Available from: http://www.abs.gov.au/websitedbs/censushome.nsf/home/seifa (Updated 27 March 2018, accessed 20 February 2019)

^b^A total of 10 indicators of financial distress were assessed (Household Expenditure Survey and Survey of Income and Housing, User Guide, Australia, 2015–16—Deprivation and Financial Stress Indicators. Australian Bureau of Statistics, 2017)

### Research team and reflexivity

The research team is collectively interested in understanding how the social determinants of health and food insecurity influence diets, with COVID-19 providing unique context for such discussions. The lead researcher (CZ) has qualifications in nutrition and dietetics, including experience conducting one-on-one dietary interviews with people from diverse ethnic backgrounds and socially and economically excluded groups. Her work has influenced her understanding of how listening to people’s lived experiences is critical to identify and address the structural barriers to healthy eating. Participants and recruiters had no pre-existing relationship with the research team and were not aware of our views and orientations to the research. Our interview guide also allowed participants to lead discussions on topics that were of primary concern to them.

### Ethics

Ethics approval was obtained by the Deakin University ethics committee prior to commencing this study (HEAG-H 122_2020). Participants were reimbursed for their time and contributions with a $AUD 50 supermarket voucher.

## Results

### Sample characteristics

Participants (n = 24) were predominantly females (n = 19), from single parent households (n = 13) and had a mean age of 41. There was approximately equal representation across Victorian Metropolitan (n = 11) and Regional (n = 13) areas, with half residing in the two lowest quintiles of area-level disadvantage and living in private rentals (n = 20). Twenty-two participants were born in Australia, two identified as Aboriginal and one indicated speaking a language other than English at home. The highest education level did not exceed a diploma (i.e., certificate for practical coursework) for eighteen of participants. Seventeen participants were unemployed or carers, with the remaining working part time or casual jobs.

All participants reported annual household incomes of less than $AUD 50,000, with ten indicating that their annual household income was less than $AUD 25,000. Prior to COVID-19, the main government income support schemes accessed by participants were NewStart (the former unemployment scheme) (n = 13) and Parenting Payments (primary income support for carers of young children) (n = 7). Following the implementation of the COVID-19 social supports, seventeen participants were accessing JobSeeker (replacing NewStart), five were receiving JobKeeper and two were receiving other supplement payments (e.g. Carers). Three-quarters of participants reported income increases at the time of interview, however, the remaining participants reported a reduction in income and/or ongoing fluctuations due to losing their job and/or becoming ineligible for Parenting and Carers Payments and transitioning to a lower JobSeeker rate. The government’s COVID-19 rent relief grant was only accessed by one participant, free childcare was accessed by four participants, and approximately two-thirds of the sample (all participants with children) reported having children at home for remote school learning in 2020. A summary of all interviewee characteristics is provided in Table 1.

### Thematic overview

The lockdowns and changes to social protection policies during the 2020 COVID-19 pandemic in Victoria did not result in consistent differences in the food security experiences of different population subgroups. As such, the themes have been pooled to reflect the collective voice of the sample. We found that it was important to give voice to participants’ broader life experiences during the pandemic (which was a difficult time for all Victorians) in order to understand where food stood as a priority and the extent to which social supports could impact change.

The temporary nature of the COVID-19 specific policy changes often meant that participants could not afford to change the way they prioritise food and healthy eating. Any additional income was generally spent on paying for housing, utility bills, and urgent (e.g., medical) expenses, with many participants still experiencing difficulty living paycheque to paycheque and being unable to save money. For a subset of participants who lost work or transitioned to lower income support rates (i.e., single parents with older children), the affordability of basic necessities, including food, became increasingly difficult. Participants’ ongoing challenges with prioritising healthy eating during COVID-19 are summarised in Table 2.

**Table 2:** Summary of key themes describing lived experiences (n = 24) with food among low-income households receiving government income support and temporary supplement payments during the COVID-19 pandemic in Victoria, Australia

**Major themes**	**Description**	**Description of sub-themes**	**Exemplar codes**
*The persistence of life’s stressors during the COVID-19 pandemic*	Despite changes to social support systems during COVID-19, participants described several competing stressors and time priorities that persisted in their daily lives. These life stressors often created a context that rendered healthy eating a less urgent priority (both prior to, and exacerbated in many ways, during COVID-19).	• Living paycheque to paycheque is hard, inflexible, and undignified now and always – there is little capacity and no savings to prioritise healthy eating in this hand-to-mouth cycle. Any additional income is typically temporary and spent on catching up with daily living expenses, which may or may not include food.	• Still had no spare money to save. • Still struggling to make ends meet. • Little savings to resist shocks like loss of income (for casual workers).
		• Co-morbid health conditions are always a financial, mental and time priority (usually prioritised before food and healthy eating).	• Ongoing struggles with disabilities. • Ongoing mental health issues. • Maintaining a healthy weight remained challenging. • Healthy eating remained a lower priority than other health issues.
		• Social and interpersonal stressors (including social support, isolation, family life) are a major priority, with lasting impacts on interactions with food and health. This was relevant both prior to and during COVID-19.	• Single parent hardship exacerbated by remote learning from home Still relied on parents to help with food and money.
*Dominant structural and financial priorities*	Participants described how they prioritised the allocation of their incomes to daily living expenses. Disposable income that was available for food was constrained by housing and utility costs, and job and education opportunities. The temporary COVID-19 Supplement payments enabled some participants to better afford necessities, but food, and consequently healthy eating, remained flexible and lower financial priorities.	• Income drives food affordability. • Temporary changes to income did not directly increase the financial prioritisation of food because income continued to be prioritised towards paying for housing, utility costs, education, and other immediate living expenses. • Most households can’t afford to purchase alcohol and do not make it a financial priority (both prior to and during COVID-19).	• Food budgets were kept low. • Food remained a secondary financial priority. • Alcohol was still unaffordable on income support. • Opportunities to earn an income and pay for food remained limited, especially during COVID-19.
		• Housing costs use up most disposable income and determine the disposable income that is leftover for food. Housing location (and where it is most affordable to live) determines which food stores and transport options are accessible. Housing remained a long-term and primary financial concern during COVID-19.	• Rent and mortgage payments always use up most of your income (housing is unaffordable). • Prioritised paying bills. • Transport options remained limited.
		• Job opportunities are hard to come by (constraining the income available for daily living expenses such as food). This was a long-term and ongoing concern during COVID-19.	• Lost job or reduced work hours due to COVID-19. • Working remains challenging when you have a medical condition. • Mothers need to raise children (reducing employment and income opportunities).
		• Education environments, costs, and skills shape opportunities for healthy eating. This remained relevant both prior to and during COVID-19.	• Remote school learning from home was hard and expensive during the COVID-19 lockdowns (kids ate more). • Navigating the social system was still hard (people don’t know how to access food charities).
*Food affordability comes before healthy eating*	Changes to food purchasing and consumption practices during COVID-19 varied. Yet, participants universally purchased foods that they perceived to be most affordable, irrespective of changes to their income and other social supports.	• Food is always a financial consideration – ‘savvy’ shopping is essential to save as much money as possible (both prior to and during COVID-19).	• Food prices continue to be a major determinant of purchases. • Continued to budget and plan by monitoring food prices across items and stores. • Continued to shop by the specials.
		• Food systems and environments promote purchases of cheap, convenient, and often unhealthy foods – especially when you’re shopping on a low budget (both prior to and during COVID-19).	• Healthy food still perceived as too expensive. • Junk food still perceived as cheap.
		• Food charities are considered important, but people prefer not to use them (both prior to and during COVID-19) due to shame, stigma, eligibility, and types of food provided.	• Only wanted to use food charities if starving.
*Snapping back policy responsibility*	Participants reflected on how social policies (and changes implemented during COVID-19) are discussed by government and the broader public – perceiving this rhetoric to be inaccurate representations of their lived experiences (both prior to and during COIVD-19). Instead of rescinding (or snapping back) policies at the first opportunity, participants described the need for governments and the whole-of-society to listen to their stories and work towards having a united voice that best serves those with firsthand experiences of food insecurity.	• Income support schemes are a lifeline for many Australians (both prior to and during COVID-19). Whilst the increased income helped people to afford basic life requirements during the pandemic, participants were aware that these payments were temporary and emphasised how the traditional rates are too low to comfortably make ends meet and prioritise healthy eating. Participants described how the reality of their lived experiences were overlooked in public and policy rhetoric – which was demonstrated by ongoing discussions of the snap back of policy responsibility during the pandemic.	• Status quo income support schemes are too low and need to be permanently reviewed.
		• Policy rhetoric around food inequity and income support (e.g. Newstart/JobSeeker) is stigmatising and inaccurate. Public perceptions that people take advantage of government income support schemes hinder attention and commitment to the implementation of effective social and food policies (both prior to and particularly highlighted during COVID-19).	• Public perceptions that people don’t want to work were elevated during the pandemic. • Public perceptions that people on income support schemes live comfortably were elevated during the pandemic.
		• Joint efforts are required across society to recognise and reduce dietary inequities over the long-term. Governments have a responsibility to listen to the stories of the people that they are serving and address their concerns (both prior to and during COVID-19).	• Governments need to do more to end poverty in Australia (an issue that was elevated in policy dialogue during the COVID-19 pandemic). • Politicians aren’t the experts in actions required to reduce social and dietary inequities (as evidenced during the pandemic). • People need to feel like valued members of society (with shared humanity also demonstrated during the pandemic).

### Theme 1: The persistence of life’s stressors during the COVID-19 pandemic

Participants collectively identified how their financial, health, and social stressors (or ‘battles’) were primary, and often urgent, concerns in their lives that ultimately superseded the prioritisation of healthy eating. This was true prior to COVID-19 and during the pandemic despite improvements or increases to government social supports.

#### Living paycheque to paycheque is a hard cycle – there is little capacity to prioritise healthy eating

All participants reflected on the hardships encountered on a day-to-day basis and how these hindered their ability to prioritise healthy eating – both financially and mentally. For most participants, this lack of fiscal flexibility did not substantially change during COVID-19 – even with the receipt of supplement payments. In the short-term, incomes were almost entirely allocated to paying for daily living expenses (i.e., rent, electricity, gas, phone, loans, school, insurance, medical bills) and did not allow them to save money, resist financial shocks, prioritise their health and wellbeing, and live with dignity. Participants described this way of living paycheque to paycheque as a ‘hand-to-mouth existence’. For one mother:

*Every dollar you have is allocated to something, and there’s not enough there as it is, but the second you get more, it’s not necessarily automatically going to be spent on something for your own wellbeing…*

*(Mother of two, Married, Metro Victoria)*

#### Co-morbid health conditions remained a major priorities (before food)

More than half of the participants described how their own health or that of a family member (and their experiences of chronic disease, disability, and/or mental illness) was a major priority – more so than food and/or healthy eating. A few participants indicated that although they had tried to prioritise improving their weight, their ongoing competing financial priorities rendered this and other self-care behaviours difficult to prioritise both prior to and during COVID-19. For example:

*…that’s always a big thing for me about losing weight and going to be a big struggle. I have sleep apnoea. I haven't been able to afford to buy a proper machine... that was actually something that I decided to do about a year ago, to work on my health a bit more. And then life just kind of got in the way, I suppose.*

(*Female, Single, Regional Victoria*)

#### Social and interpersonal stressors have lasting impacts on interactions with food and health

Adding further complexity to their daily lives, participants described social and interpersonal stressors, often through family dynamics and living arrangements. These included single parent hardship (i.e., one income, women often left to raise children, history of partner/domestic abuse), divorce, sacrifices for children (and often feeling unable to live up to parenting expectations), intergenerational poverty, and caring for other family members and animals. Such social factors contributed to daily experiences with hardship and made it difficult for participants to make their health a financial and/or mental priority – even with the implementation of several COVID-19 specific social supports. One regional dwelling mother said:

*I am a single mum of three, and currently I live at home with my two parents and my elderly grandmother. So, we live in a house that has seven people. My dad is a carer for my grandmother, my mum is currently also on JobSeeker. And it’s crazy, it’s really difficult. We’ve almost lost our home… my parents have almost lost their car, we - both myself and my mum, we are both currently sitting in the crack of being in chronic illness but not quite on DSP (Disability Support Pension).*

*(Single mother of three, Regional Victoria)*

The importance of social support from family and friends – through either the provision of food or money – was seen to be critical to helping participants and their families get by both prior to and during COVID-19. This idea was often reported alongside statements of shame whereby participants often recollected how they did not want to, and should not have to, rely on others for such help:

*I suppose at my age it’s really embarrassing because my mum helps us a lot. So she does a lot of grocery shopping for us… I'm not scared we’re ever going to go hungry because my mum’s amazing, but I can’t afford that. So if I didn't have my mum who works fulltime and has no huge bills, she already owns her own house and stuff, then I don't know what we would do.*

*(Mother of two, Married, Regional Victoria)*

In contrast, some participants indicated that such social support was not available to them and exacerbated feelings of isolation during COVID-19. This resulted in low motivation to cook, skipping meals, and increased reliance on purchasing take-away foods if the money was available (discussed in further detail in Theme 3).

### Theme 2: Dominant structural and financial priorities

There was no consensus on how increases to government social supports (namely income supports) affected expenditure on food. Many families prioritised using their additional income to pay for other basic living expenses; for example, to get ahead on bills rather than altering their food budgets.

#### Income drives food affordability

The income available to unemployed Australians receiving income support prior to COVID-19 (i.e., NewStart) was universally deemed to be inadequate for surviving, with participants describing how they would ‘just scrape by.’ As participants battled with their competing financial priorities, food budgets were perceived to be the most flexible part of this income and reported to be as low as $AUD 20 for some individuals and $AUD 80–100 per week for three to four person families. A few participants recognised this as income-driven food insecurity.

*…food itself, I feel that food itself is not that expensive. It’s more about that we just can’t afford it. If you’re on a working income, food is not expensive at all.*

*(Female, Single, Regional Victoria)*

Recognising the temporary nature of the government COVID-19 Supplement payments, many participants kept to their usual budgeting practices. As described by one father:

*I stocked up where I could. My pantry's always got food in it. I try and do a big shop at least every two or three months, where I spend everything I get just on food. I don't think there's anything different I've been doing.*

*(Single father of two, Regional Victoria)*

Some participants reported using their income supplements to buy higher quality fruits, vegetables, meats, and dairy products compared to the more typically sourced frozen varieties and ‘cheap carbs’. Other participants indicated that they increased their purchases of take-away foods. However, due to their extremely low food budgets prior to COVID-19, most participants indicated that they rarely ate takeaway foods. For those families who experienced a decrease in income during the COVID-19 pandemic, they reported eating whatever was cheap and available and relying on family or friends for meals. The importance of income changes for food purchasing was highlighted by a new single mother:

*At the moment I can prioritise healthy foods due to the Coronavirus Supplement, plus the increase because of being a single parent now, and the fact that my brother is chipping in. I can get fruit and stuff, but before all this, it was just basically whatever we could afford to make a cheap meal.*

*(Single mother of one, Metro Victoria)*

Most households also indicated that they could not afford to purchase alcohol, both before and after their income increased with COVID-19 Supplements; with most of their food budgets always prioritised towards feeding their families. One father described alcohol as a ‘luxury’ item:

*My wife doesn’t really drink a great deal; I enjoy a drink. I, basically, stopped buying alcohol because, in my mind, that was a luxury item that we didn’t really need.*

*(Father of one, Married, Regional Victoria)*

#### Housing costs are a major determinant of disposable income for food

Issues with housing affordability persisted throughout the COVID-19 pandemic. Participants all agreed that most of their income was always prioritised to pay for housing – either rent or mortgage payments. A married mother of two articulated these priorities:

*Well, it’s an essential thing, we must have food to survive. We have to have a roof over our heads too, but I think if we’re going in order of priority for financial – for managing financially, I guess our rent always comes first because we must have somewhere to live first of all and then food and then bills.*

*(Mother of two, Married, Metro Victoria)*

Whilst existing government rent assistance was seen to be helpful, some participants still reported that rental payments were a long-term financial constraint, constituting as much as 90% of their fortnightly income. This reiterated how money for food is a lower and more flexible financial priority. One mother advocated that:

*… no one should be disadvantaged by the food that they have to eat, to be able to live in their house… You have to choose between food or your house, or your warmth, or the internet for your children because they have to do schooling as well. Like, you shouldn't have to choose that. You should be able to do it all. You know, we don't live in a third world country, but sometimes parts of it feel like it is.*

*(Single mother of three, Regional Victoria)*

In addition, concerns were voiced about the constant insecurity associated with rental payments and the low-quality housing options. A few participants who were homeowners suggested that they also did not receive enough income support to pay for housing, prior to and during the COVID-19 pandemic.

Housing location was further described as a key determinant of participants’ abilities to access food, both in terms of the proximity of grocery stores and access to transport (which was typically limited). A few participants indicated that they used their additional COVID-19 Supplements to access their preferred grocery stores (usually the major compared to discount supermarkets) or fix their car. Whilst participants suggested that actions were required to improve housing affordability, only a few tangible solutions were identified; including investment in social housing, providing vouchers and higher subsidies for utilities and services or making it easier for people on low incomes to access existing support schemes.

#### Job opportunities remained hard to come by

Except for one participant, all participants reported precarious work situations (unemployed/casual/part-time). Many reported either losing their jobs or a reduction in working hours during the COVID-19 pandemic. Without the government income support payments, participants indicated that they would have reduced their food budgets – which was the case with participants who experienced a decrease in income:

*My income did drop. I lost over half of my hours every week before obviously… I think I was doing 20 hours a week, I dropped to eight hours, which is my absolute minimum. So that, I was on, I think, about $170 a week, rather than almost 400. I would not have been able to cover my rent, my food barely, and my utilities, and that would have been about it. There would’ve been nothing.*

*(Single mother of two, Regional Victoria)*

It was also emphasised that there were limited opportunities for permanent, ongoing work – especially in Regional Victoria. Furthermore, participants described how they were not in positions to work due to health conditions or having to care for family members (including raising children). Whilst some participants reported generating income by selling valuables during COVID-19, these challenges with work were largely unimproved by the COVID-19 specific social supports.

#### Education environments, costs, and skills shape opportunities for healthy eating

Participants who had children at home during the COVID-19 lockdowns collectively described remote school learning as hard. Concerns were expressed by parents regarding the future implications of learning from home on their child’s development and future. Although some families kept to their food budgeting and planning practices (including planned meals for children), other families described how it became more expensive to feed and educate their children as they were learning from home. These varied experiences were reflected by two mothers:

*We were very good at staying to a simple routine and sticking to it so we had meals at the same time each day so that didn’t really change much.*

*(Mother of two, Metro Victoria)*

*…my kids basically ate me out of house.*

*(Single mother of five, Regional Victoria)*

Even though the four participants who received free childcare at the beginning of the COVID-19 pandemic expressed support for this policy action in general, participants generally suggested that their pre-COVID-19 childcare subsidies were substantial and adequate. As such, little additional savings were accumulated during the pandemic, and these were typically repurposed to pay for other daily living expenses (based on the financial priorities described above).

A few participants suggested that nutrition education and cooking classes could improve their ability to eat healthy, and that education on how to navigate the social system could also be beneficial. Nonetheless, most participants did not indicate a deficit in knowledge to be a key driver of their food behaviours – with one indicating that this belief was condescending:

*It's condescending that you are not treating people like adults, that somehow people on unemployment are stupid. We’re not. We are not stupid at all. In fact, I’ve got three lots of university qualifications. I’m not stupid... It has to be just a subtle way of saying that eating fruit and veggies is cheaper than buying takeaway, which some people don’t realise is the case… It comes down if you have no money… people will not be able to buy what you know they need to eat anyway.*

*(Female, Divorced, Regional Victoria)*

### Theme 3: Food affordability comes before healthy eating

#### Food is always a financial consideration – you need to save as much money as possible

Participants overwhelmingly conveyed the idea that despite their best efforts to eat healthy, food was always a financial concern and they could only eat what their income allowed them to afford. Consequently, food and beverage prices (and pricing discounts) were pertinent determinants of a household’s food choices** –** food choices and purchases that are typically made just to ‘get by’. Food and beverage prices were deemed to be critical both before and during the COVID-19 pandemic. To manage their low food budgets, participants reported high levels of food pricing knowledge and ‘savvy’ budgeting and shopping skills, including monitoring price promotions and their total expenditure. Many families maintained these budgeting and planning practices throughout the COVID-19 pandemic as they were generally aware that increases in income from COVID-19 social supports would eventually be rescinded. Nevertheless, participants indicated that they were less concerned about running out of money for groceries when they had the extra income. One participant explained how:

*The bills were paid. I went to get groceries and I didn’t have to feel fear at the checkout thinking I’d have to put food back.*

*(Mother of one, Divorced, Metro Victoria)*

#### Food systems and environments promote purchases of cheap, convenient, and unhealthy foods and beverages (especially when you’re shopping on a low budget)

Participants described how supermarket price promotions (i.e., discounts or specials) influenced their food and beverage purchases – including stockpiling price promoted products with a long shelf life to ensure that they would never run out of food. Price promotions and food prices would also influence where participants would shop – with discount supermarkets ALDI and Not Quite Right (NQR) frequently identified as the cheapest retailers. Participants perceived unhealthy foods and beverages to be more affordable and aggressively discounted than healthy foods such as high-quality meat, fruit, and vegetables. Participants subsequently based their food purchases around ‘cheap carbs’ such as pasta, rice, noodles and cereals. Some reflections were also provided on how food and beverage prices are manipulative for people on low incomes and how the in-store and online supermarket environments make people ‘impulse buy’, especially when shopping with children. According to one father’s experience:

*Well, Doritos are always half price. I’ll just buy those and that could be lunch because I can’t afford to buy anything healthy. And it shouldn’t be like that. And I think that’s a real issue…the same when you talk about catalogues too, is that the front and back pages, they are all just shit food and not stuff that you should be buying. They’re the stuff that’s half price.*

*(Single father of one, Regional Victoria)*

Few changes to participant interactions with food systems were observed during the COVID-19 pandemic, with several participants suggesting that price increases had occurred for some healthy staple options (lean meats, fruits, and vegetables).

#### Food charities are considered important, but people prefer not to use them

Food charities were commended for the service they provided, including during the pandemic, often accessed by participants to obtain fruits and vegetables, and recognised to reduce food system waste. With the COVID-19 Supplements, participants described mixed use of food charities, with some indicating that they continued to access these services and others reporting decreased use. Despite their hardship, participants often indicated how they would only use food charities if they were ‘starving’, perceiving other families to be in greater need, issues with eligibility, low awareness of how to access them, and some dislike for the foods offered. One mother shared her experiences with food charities:

*Prior to COVID, yes* <<*to accessing food charities*>>*. I go through waves almost where I would use them – sounds bad; use them. No, I would go to them, utilize them, off and on for a month or two, by which point I would've managed to get myself back into an OK position where I try not to use them. Because whenever I do go there I feel like I'm taking food from someone else who really needs it. So, I would try not to. But the very first thing you do when you walk in the food bank is you go to the fruit and veg section.*

(*Mother of two, Divorced, Regional Victoria*)

### Theme 4: Snapping back policy responsibility

#### Income support policies were a lifeline for many Australians

Participants whose incomes increased during COVID-19 described the government actions as a lifeline for themselves and many Australians in similar situations. The pre-COVID unemployment support scheme (Newstart) and the temporary nature of the COVID-19 increase to this payment (JobSeeker and JobKeeper COVID-19 Supplements) were collectively perceived as negative, with suggestions that the government’s pre-COVID income supports were not sufficient and needed to be permanently increased. The Government’s Carers, Parenting and Disability Support payments were viewed more favourably due to their higher rates and additional flexibility to cover basic living expenses. Several participants had submitted applications for the Disability Support Scheme due to chronic illnesses that were reported to be inadequately recognised by the scheme. There was consensus that government income support schemes allow people to, at best, afford their basic financial needs – with food (but not necessarily healthy food) being one of these. As such, concerns were expressed about the COVID-19 Supplements being rescinded:

*You can’t take away something that people had and then expect them to still be happy about it. Here you have this little bonus, now go back to eating dirt for the next month.*

*(Female, Divorced, Empty Nester, Regional Victoria)*

*I’m grateful that they stepped up, and they helped and we’ve got pretty lucky in Australia. But it is time for a little bit of improvement as well in regards to welfare… living below the poverty line isn’t fun.*

*(Mother of two, Single, Regional Victoria)*

#### Policy rhetoric around food insecurity continued to be stigmatising and inaccurate

Irrespective of any additional COVID-19 Supplements, participants described constant financial struggles. Financial struggles included the affordability of food and subsequent stigma associated with food poverty; for example, being seen as ‘bottom scrapers’ and societal expectations to ‘live within your means.’ Most participants described how the common perception (which was elevated during the pandemic to suggest) that people take advantage of government benefits by being financially irresponsible, living luxuriously, and spending their incomes on unhealthy/harmful activities, was inaccurate.

A desire to work was also expressed but participants indicated that job opportunities were limited. Indeed, it was conveyed how people experiencing socioeconomic disadvantage often do not want to receive ‘handouts.’ Five participants extended this perception to negative stigma associated with cashless debit cards, which are compulsory for recipients of government support payments in some areas of Australia. Reflections were further provided on the disconnect between these lived experiences and public and policy rhetoric (which was common during the COVID-19 pandemic) – with the latter depicting people on income support schemes as lazy and needing to budget better. One mother described the stigmatising impacts of such rhetoric:

*I read on Facebook all the posts about the new things they're bringing in … and it all come back to welfare. ‘Everyone’s helping them and not the working class.’ The comments are horrible, but every cent helps that they do, and if you're lucky enough to keep your job – like if I didn't have to give up my job – I had a great job… It was amazing and I chose this life. Like it wasn’t really a choice, but my daughter wouldn't be here if I didn’t give up work. No one’s interested in hearing your story. I don't think you’ll ever get rid of the stigma at all.*

*(Mother of two, Married, Regional Victoria)*

Overall, participants conveyed the idea that they were doing their best to get by but did not feel as though their voices and experiences were being seen or heard by society – especially by decision makers. This rendered them doubtful that the COVID-19 social policy changes would be implemented to improve their situation and reduce the stigma associated with food insecurity in the long-term. This notion was expressed by one participant’s direct call to the Prime Minister:

*Give him* <<*the Prime Minister*>> *three months to live on what we live on and then put him in public housing…because I can tell you now, he’s not living on what we’re living on and where we’re living.*

*(Single mother of three, Regional Victoria)*

#### Joint, ongoing efforts are required across society to reduce food inequity

The rapid introduction of COVID-19 Supplements was perceived to be one way that the government showed genuine concern for people experiencing financial hardship and food insecurity. To combat the inaccurate and stigmatising policy rhetoric around government income support, participants suggested that there was a need for governments (across all levels) and the whole-of-society to continue to listen to the evidence, experts, and people’s lived experiences – not as a tokenistic gesture – but in a way that makes people who receive low incomes feel like valued members of society and part of a process that informs action. It was also suggested that governments and other influential members of society should cease ‘hiding behind privilege’ and making excuses for inaction on the social determinants of food inequity (i.e., income, housing affordability, job opportunities). Participants indicated that a country like Australia has the resources to end poverty:

*I remember a few years ago… Some woman in parliament said, ‘Oh, people in welfare, they just need to budget better.’ And I was so angry I wrote her a letter. Basically saying, ‘Oh, okay, well here’s my budget. Income $260, rent $250. Okay. And then break down that $10 for all the other costs of living.’ And what I got back from her was a three-page letter just justifying her position, and it made me even more angry… it was so arrogant and so evident that they had no [censored] clue, excuse my language, about what it’s like to be on this end of the financial scale.*

*(Female, Single, Regional Victoria)*

In addition to governments, other actors, including the private sector (i.e., banks, supermarkets), community organisations and schools, were recognised as important sectors to reduce food inequity. Whilst recommended actions varied across sectors, participants indicated that the COVID-19 pandemic potentially increased public awareness of how food inequity was everyone’s responsibility, and there was a need for one voice (publicly and politically):

*…it’s a blame game. It’s all about things that have gone wrong and whose fault it is and things like that, rather than ‘Hey, let’s see how we can help people. These are the people we work for. Let’s make sure their lives can be a little less difficult.’*

*(Single mother of two, Regional Victoria)*

## Discussion

At the end of 2020, one month after Victoria exited a five-month state-wide lockdown in response to the COVID-19 pandemic, 24 Victorians who were receiving government income supports (prior to COVID-19) described few differences in their food-related experiences. This was surprising and contrary to our hypotheses given the unprecedented changes to the social determinants of food insecurity (i.e., most receiving increased incomes through additional COVID-19 Supplements). The temporary nature of these policy changes typically meant that any additional income was prioritised towards paying for housing, utility, and other urgent expenses, rather than on food or healthy eating – which were still perceived to be lower and more flexible financial priorities. Many participants described entrenched challenges with prioritising healthy eating in the context of constant difficulties associated with living paycheque to paycheque, and food systems that promote purchases of cheap, convenient, and unhealthy foods and beverages. Participants consequently perceived the elevated policy rhetoric around people receiving government income support (and therefore experiencing health inequities) to be inaccurate and shaming – often misrepresenting their lived experiences, both prior to and during the COVID-19 pandemic.

Below we outline key lessons learned from our study (summarised in Table 3) and their alignment with existing theories and evidence. Our research firstly builds upon our understanding of how policies can act on the social determinants of health and health inequities in the real-world (World Health Organization, 2008). With few empirical opportunities available to understand how social policies can be used to reduce food insecurity, our empirically derived lessons and lived experience narratives are important to broaden the policy debate beyond the overreliance on food charities in Australia and many other countries (Pollard & Booth, 2019). Lessons one and three explicitly demonstrate this idea – challenging the inadequate policy efforts of non-health sectors to engage in and address key determinants of food insecurity until the COVID-19 pandemic in Australia. Lesson two subsequently challenges Australia’s inadequate policy focus on comprehensively addressing the unaffordability of healthy diets for low-income households (an issue that has been quantified previously (Zorbas et al., 2022) but that the pandemic has thrown into the spotlight). Finally, we argue that the COVID-19 specific policy response demonstrated the recognised responsibility of governments for addressing food insecurity – including by driving appropriate policy and public rhetoric that reflects lived experiences of the social determinants of disadvantage and enables long-term, structural policy progress (Backholer et al., 2014).

**Table 3:** Key lessons learned from our study and recommendations to reduce inequities in population diets

***Lessons***	*Overarching lessons:*	*Additional relevance of this study and Australia’s COVID-19 specific policies:*
**Lesson one**	Long-term nutrition-sensitive policy actions^a^ are needed on the upstream structural drivers of food insecurity	• Theme 1 from our study highlights the need to recognise that the lives of people who receive government income supports are often dominated by major and ongoing stressors. This means that purchasing and consuming healthy foods (i.e., preventive health) are not major priorities in the short-term. Thus, short-term policy changes such as those implemented during COVID-19 will be limited in their diet-related health impacts. Long-term, system-wide policy changes will be needed to collectively reduce the life stressors of people who experience social disadvantage and make healthy diets easily accessible and affordable for these groups • Theme 2 provides narratives that reflect the unequivocal need to work towards cross-sectoral actions to address the social determinants of food insecurity (especially by improving incomes, housing affordability, and job opportunities). These have traditionally been addressed in a siloed fashion and considered out of scope by governments and other stakeholders. Australia’s COVID-19 specific policies demonstrated that it is possible (and should be a long-term priority) for governments to address public health issues by acting on the social determinants of health
**Lesson two**	Nutrition-specific policy actions^b^ are needed to address the price and affordability of healthy and unhealthy diets	• The price of healthy compared to unhealthy foods and beverages is not comprehensively addressed by governments in many countries (Zorbas et al., 2020b). Governments should begin to explore comprehensive and equitable food pricing policy strategies that enable the affordability of healthy over unhealthy diets (include subsidies, taxes, and regulations of retail pricing strategies such as price promotions)
**Lesson three**	Intersectoral policymaking processes and rhetoric need to be more inclusive of lived experiences of food insecurity	• Governments must continue to recognise their responsibility for committing to long-term actions to reduce food insecurity. Part of this responsibility includes driving public rhetoric that reflects lived experiences of social disadvantage and the social determinants of food insecurity. The Australian Government’s social policy response during the COVID-19 pandemic demonstrated how conflicting rhetoric is likely to impede appropriate policy progress

^a^Nutrition-sensitive policy actions are defined as non-health sector actions that directly affect the social determinants of health (e.g. income support, housing affordability, employment) (Pollard & Booth, 2019)

^b^Nutrition-specific actions are those that change daily living conditions to directly affect food behaviours and security (e.g. subsidies on healthy foods, taxing sugar-sweetened beverages, food labelling) (Pollard & Booth, 2019)

The findings collectively highlight the idea that a radical rethink is needed by all stakeholders (including academics, policymakers, and practitioners) to prioritise, create, and adopt policies that meaningfully impact the lives of people who experience chronic poverty. Approaches to decision-making are needed that account for (rather than overlook or underestimate) the complexity of these real-world experiences and challenge underlying power imbalances and traditional notions of expertise.

### Lesson one: Long-term nutrition-sensitive policy actions are needed on the upstream structural drivers of food insecurity

Our findings are supported by existing literature on Australian families’ lived experiences with socioeconomic disadvantage (Daly et al., 2018; McKenzie et al., 2019; Phillips et al., 2021). In 2013–14, several studies were conducted to understand how single mothers experienced decreases in government income support after transitioning from Parenting Payments ($720 per fortnight; ineligible once their youngest child turns eight) to a lower rate for the unemployment support scheme (Newstart) ($558; below the poverty line) (McKenzie & McKay, 2017, 2018; McKenzie et al., 2019). These studies outline similar stressful experiences of mothers who needed to balance their limited finances – with housing and utility bills prioritised first, followed by transport, education, and food (the negotiable part of the budget); having to forego social activities and medical expenses; demonstrating adept budgeting and shopping skills; and the importance of social support and asking for help with money and food (McKenzie & McKay, 2017, 2018; McKenzie et al., 2019). Our themes *‘The persistence of life’s stressors during the COVID-19 pandemic’* and *‘Dominant structural and financial priorities’* additionally demonstrate that short-term government policies were potentially inadequate in addressing these persistent experiences with food insecurity. Rather than focusing on individual behavioural adaptations to living on low incomes, we show that sustained commitment to stronger policy actions is likely to be needed to disrupt the structural deterrents to purchasing and consuming healthy diets.

Reframing nutrition as a structural and systemic policy issue is critical to ensure that diet-related health and illnesses are considered in the context of people’s lived experiences. Such reframing underscores the need to implement policy actions outside of the health sector (i.e., nutrition-sensitive actions) (Pollard & Booth, 2019). The development of Western Australia’s area-level Food Stress Index is one example of how intersectoral collaborations (i.e., governance, food relief, community organisations) may support an increased and sustained focus on structural social policy actions to reduce food insecurity (Pollard et al., 2021). The Food Stress Index clearly shows how household incomes and housing expenses should be recognised as major determinants of inequities in diet-related health by policymakers and all stakeholders (Landrigan et al., 2018).

Almost overnight, the 2020 Australian COVID-19 Supplements substantially reduced poverty among those receiving unemployment support (JobSeeker), from 88 to 26% (Phillips & Narayanan, 2021). At the beginning of 2021, the JobSeeker Supplement (additional $550/fortnight at its peak) was completely rescinded but the Government increased the base rate of this unemployment support scheme by $AUD 50/fortnight – moving Australia from the lowest to second lowest income support rates among the OECD (compared as a percentage of average national income) (Coates & Cowgill, 2021). Even with the current $AUD 50/fortnight increase, the Government’s decision to rescind the COVID-19 Supplements is estimated to return poverty rates back to 85% among people receiving unemployment supports (Phillips & Narayanan, 2021). Sustained, rather than temporary, government investment in adequate income support and affordable housing (alongside other social protection measures) is core to the health and wellbeing of our societies – especially when one in five Australians depend on social protection schemes (Temple et al., 2019). The COVID-19 pandemic uniquely demonstrates that it is possible for the Australian Government to make long-term policy investments to address public health issues by acting on the social determinants of health.

### Lesson two: Nutrition-specific policy actions are needed to address the price and affordability of healthy and unhealthy diets

Our study reiterates findings within the broader literature that suggest food prices and their affordability are one of the most important levers to equitably promote healthy population diets (both pre- and post-COVID-19) (Zorbas et al., 2018). Evidence thereby indicates that comprehensive, nutrition-specific pricing policies are required to increase the affordability of healthy options and reduce the affordability of unhealthy options (Pollard & Booth, 2019; Zorbas & Backholer, 2019; Zorbas et al., 2020b). Such policies should include restricting price promotions for unhealthy foods and beverages and incentivising price promotions on healthy options, taxing sugar-sweetened beverages and unhealthy foods, improving healthy food subsidies, and nutrition-focused food banking (Pollard & Booth, 2019). Participants widely described their price-sensitivity through the importance of purchasing price-promoted foods (which were typically thought to be less healthy, ‘cheap carbs’) irrespective of any change to income. These perceptions suggest that price promotions have the potential to change the purchasing patterns of those with low incomes – building upon our previous research showing how price promotions are widely available and purchased (Riesenberg et al., 2019; Zorbas et al., 2019, 2020a, b). In 2017, we found that 50% of New Zealand household food and beverage purchases were price promoted – with low-income households purchasing significantly more price promoted items (52%) than high-income households (46%) (Zorbas et al., 2020a, b).

In the UK, nutrition-specific policy actions will be enacted to restrict volume-based price promotions on unhealthy foods and beverages as part of a more comprehensive strategy to create food environments that promote healthy, over less healthy, food and beverage purchases (UK Government, 2020). Yet, in most other countries, including Australia, policy actions to address the influence of food and beverage prices and reduce inequities in diet-related health have been slow (Zorbas et al., 2020b, 2021). In Australia, existing fiscal mechanisms (i.e. the Goods and Services Tax) are available to increase the tax on unhealthy foods and beverages, whilst maintaining the tax exemption on healthy options (Landrigan et al., 2017). However, political will to act is lacking and is likely to have left many Australians vulnerable to food insecurity and dietary risks during the COVID-19 pandemic. As such, our study suggests that the COVID-19 pandemic and the associated policies provided a novel opportunity to highlight the inadequate response of the Australian Government to date in addressing food security for all. Furthermore, whilst our participants did not appear to be impacted by changes to food prices or food supply shortages during the COVID-19 pandemic, ongoing research is required to inform the design and implementation of equitable and resilient food systems into the future.

### Lesson three: Intersectoral policymaking processes and rhetoric need to be more inclusive of lived experiences of food insecurity

A key contribution of our study is the finding that Australians continue to feel ashamed, unheard, and hopeless because of the dominant public and political rhetoric around social, economic, health, and food inequities. This rhetoric and neoliberal framing of ‘dole bludgers’ became elevated as the pandemic progressed and suggests that people receiving government income support take advantage of ‘welfare benefits’ and are too ‘lazy’ to work (Archer, 2009). In contrast, our lived experience data show that people receiving government income support, even with temporary increases in support, struggle to afford basic needs (including food) and do not ‘choose’ luxurious lifestyles. Based on these findings, the perceptions, voices and lived experiences of single mothers and people with chronic health issues and/or disabilities do not appear to be heard by Australian policymakers. Our findings might also suggest that conflicting policy rhetoric (such as that which was elevated during the pandemic) may impede appropriate social policy progress. Our previous research supports the notion that government rhetoric is not equity-oriented with respect to addressing population diets in high-income countries (Zorbas et al., 2021). We found that equity was only a surface-level consideration in national nutrition policy strategies, with actions focused on changing individual-level behaviours rather than structural drivers of diet-related health inequities (Chung et al., 2021; Zorbas et al., 2021).

To reframe rhetoric around diet-related health inequities, emerging evidence indicates that there is a need for all sectors to listen to and act upon the voices of those who experience social or economic exclusion (Centre for Public Impact-A BCG Foundation, 2020). Indeed, NGOs, researchers and governments are increasingly recognising that ***“People are not hard to reach – they have voices, views, and great ideas too – but they are seldom heard.”*** (*Laura Seebohm, Executive Director, External Affairs at Changing Lives; Nadine Smith, Director of Centre for Public Health Impact*) (Centre for Public Impact—A BCG Foundation, 2020; Phillips et al., 2021). Listening has the power to build relationships and empathy, allow people to feel seen and heard, increase a sense of belonging and purpose, encourage ongoing participation, lead to learning and new insights, and motivate action (often to help others) (Centre for Public Impact—A BCG Foundation, 2020). To design and deliver more inclusive and impactful food policies, co-creation methodologies that challenge traditional power imbalances and assumptions in decision-making, by involve listening to and working with priority populations as equal partners (alongside policymakers, researchers, public/private industry stakeholders), are likely to be important (Food Secure Canada, 2011; Leask et al., 2019). Additional research is required to understand best practice approaches to using co-creation to strengthen equitable food policymaking. In the meantime, governments should adopt existing tools and equity impact assessments to ensure that policy actions reduce, rather than exacerbate, inequities.

### Strengths and limitations

Our study offers novel insights into experiences of real-life policy actions on the social determinants of food insecurity during the COVID-19 pandemic in Australia, including changes to government income support payments (which seldom occur in many high-income countries). Whilst our sample size and interview durations enabled us to collect rich data, our methods may have benefited from a stronger ethnographic orientation to interrogate lived experiences further, including within key population groups, which the lockdown restrictions impeded at the time of the study. These methods should continue to be used to unpack the intersectional lived experiences with food insecurity and inform more targeted policy recommendations in the future.

## Conclusions

The COVID-19 pandemic prompted the Australian Government to introduce additional social supports and provide significantly higher temporary incomes for many Australians experiencing hardship. Yet, the temporary nature of these income supports may not have changed experiences with major determinants of food insecurity among low-income households, with food remaining a lower and more flexible financial priority compared to other structural costs such as housing. Leadership by governments and all stakeholders is critically required to listen to priority populations’ lived experiences of food insecurity and support the implementation of tailored policy actions that address key structural drivers of population diets into the future.

## Supplementary Information

Below is the link to the electronic supplementary material.

Supplementary file1 (DOCX 22 KB)
