# Supplementary material for: Shifting the social determinants of food insecurity during the COVID-19 pandemic: the Australian experience
Source: Food Secur. 2022 Sep 17;15(1):151–70. doi: 10.1007/s12571-022-01318-4 (PMC9483265; doi:10.1007/s12571-022-01318-4)
Supplement: Supplementary file 1 — Supplementary file1 (DOCX 22 KB) [file 12571_2022_1318_MOESM1_ESM.docx]

**SUPPLEMENTARY INFORMATION:**

**FOOD SECURITY**

**Lifting the silence on lived experiences of food insecurity among households receiving government income support: an Australian qualitative study during the COVID-19 pandemic**

Christina Zorbas^1^, Jennifer Browne^1^, Alexandra Chung^2^, Anna Peeters^1^, Sue Booth^3^, Christina Pollard^4^, Steven Allender^1^, Anna Isaacs^5^, Corinna Hawkes^5^, Kathryn Backholer^1^

*^1^Global Obesity Centre, School of Health and Social Development, Institute for Health Transformation, Faculty of Health, Deakin University, Geelong, Australia*

*^2^School of Public Health and Preventive Medicine, Monash University, Melbourne, Australia*

*^3^College of Medicine and Public Health, Flinders University*

*^4^School of Population Health, Faculty of Health Sciences, Curtin University*

*^5^Centre for Food Policy, School of Health Sciences, Division of Health Services Research and Management, City, University of London*

**CORRESPONDING AUTHOR**:

Christina Zorbas

[c.zorbas@deakin.edu.au](mailto:c.zorbas@deakin.edu.au)

**Table S1: Overview of COVID-19 policy changes affecting the social determinants of diet-related health in Victoria, Australia (1)**

| **Social determinant of health** | **Policy change (Victoria/Australia)** | **Relevance to diet-related health** |
| --- | --- | --- |
| **Income** | - **Income support:** Australians received a $550 JobSeeker Supplement payment fortnightly if they are eligible for income support schemes during COVID-19 (Australia) (2) | - Potential to change the amount of disposable income available for food and beverage purchasing |
| **Employment** | - **Wage subsidies:** JobKeeper provided $1500 fortnightly payments to employees via employers that record 30% revenue losses (Australia). This approximately double the previous income support scheme (NewStart). An additional one-off $750 Economic Support Payment was also provided on March 31 (3) | - Potential to change the amount of disposable income available for food and beverage purchasing |
| **Housing** | - **Rent relief grant:** one off $2000 rent payment for Victorian households experiencing rental hardship (defined as having <$5000 in savings, using at least 30% of your income on rent, and earning less than $1903/week); 6-month ban on increases in rent (Victoria) (4) | - Potential to reduce pressure on food and beverage budgets |
| **Early childhood and education** | - **Free childcare** (Australia) (5) - **Learning from home** (Victoria) (6) | - Free childcare may change the amount of discretionary funds available to households (within the context of other changes to income) - An increase in learning from home may change household eating patterns and the home food environment |

**REFERENCES**

1. Australian Government. Department of Education, Skills and Employment. Coronavirus (COVID-19): Responding to the Coronavirus (COVID-19) pandemic. [Updated 5 Oct 2021; accessed 6 Oct 2021] Available from: <https://www.dese.gov.au/covid-19>
2. Australian Government. Fact Sheet: Economic Response to the Coronavirus – Income support for individuals. [Updated 14 April 2020; Accessed 28 May 2020] Available from: <https://treasury.gov.au/sites/default/files/2020-04/Fact_sheet-Income_Support_for_Individuals.pdf>
3. Australian Government. Fact Sheet: Economic Response to the Coronavirus. JobKeeper Payment – Information for employees. [Updated 20 April 2020; Accessed 28 May 2020] Available from: <https://treasury.gov.au/sites/default/files/2020-04/Fact_sheet_Info_for_Employees.pdf>

State Government of Victoria. Department of Families, Fairness and Housing. Coronavirus (COVID-19) rent relief grant. [Updated 30 Sept 2021; Accessed 28 May 2020] Available from: <https://www.housing.vic.gov.au/help-renting/rentrelief>

1. Parliament of Australia. COVID-19 Economic response – free child care. [Updated 6 April 2020; Accessed 28 May 2020] Available from: <https://www.aph.gov.au/About_Parliament/Parliamentary_Departments/Parliamentary_Library/FlagPost/2020/April/Coronavirus_response-Free_child_care>
2. State Government of Victoria. Department of Education and Training. Learning from home information for parents. [Accessed 28 May 2020] Available from: <https://www.education.vic.gov.au/parents/learning/Pages/home-learning.aspx>

**Table S2: Australian Bureau of Statistics Financial Stress Indicators (24) – screening questions for participants**

|  | I sought assistance from welfare/community organisations? |
| --- | --- |
|  | I had to go without meals? |
|  | I have had to seek financial help from friends or family? |
|  | My household spent more money than it got (over the past 12 months)? |
|  | I would not have been able to raise $2,000 in a week for something important? |
|  | I was not able to afford to heat my home? |
|  | I was not able to pay electricity, gas or telephone bills on time? |
|  | I was not able to pay car registration or insurance on time? |
|  | I have pawned or sold something to raise money for essential items? |
|  | None of the above apply |

**Table S3: Overview of interview guide**

| **PART 1: Introduction** | **1a.** Thinking about your life now during the COVID situation and into the future – what are some of your major concerns? |
| --- | --- |
| **PART 2:**  **Policy changes due to the COVID-19 pandemic and lockdown restrictions** | **2a.** Thinking about what life was like before the COVID lockdown measures were introduced in March (say in January or February), how did income support payments affect you and your family’s income?  **2b.** And thinking about your life during the COVID lockdown and with restrictions easing now, how have income support payments like JobSeeker and the Coronavirus Supplement affected you and your family?  **2c.** How did you spend the one-off Coronavirus Support Payment of $750?  **2d.** We’re really interested in exploring changes to the way you have been eating or shopping now. Can you tell me how the COVID lockdown since March has impacted the way you or your family buy and eat food?  **2e.** Following on from this, has the money from JobSeeker and the coronavirus supplement since March impacted the money available to you or your family buy or eat food? Could you tell me a bit about this?  **2f.** Still thinking about COVID and food, if there were no changes to the JobSeeker payments – and things were the same as January or February before the COVID lockdown – how do you think you and your family would have gone with buying food during this time?  **2g.** Switching focus a bit to think about COVID and alcohol now – can you tell me if JobSeeker has changed the money that is available to you or your family to buy and drink alcohol since March? |
| **PART 3:**  **Policies for the future** | **3a.** Thinking about your life now during the COVID situation – it looks like things will have to go back to normal and that policies or strategies like JobSeeker, JobKeeper, rental relief grants, and free childcare will only be around for 3 to 6 months. If you were the Prime Minister – what would you do to make it easier for people doing it tough to buy and eat foods?  **3b.** How can people (like charities, community organisations, local and state governments) help families like yours to eat and be healthy during this time? |
